# Conserved Core and Species-Specific Signatures in the Milk Exosomal microRNA Targetome: A Preliminary Comparative In Silico Analysis of Human, Cow, Goat and Donkey Milk

**DOI:** 10.3390/ijms27156952

**Published:** 2026-08-02

**Authors:** Maksym Zoziuk, Abel Dafogo Djibagaou, Alessandro Terrinoni, Dimitri Koroliouk, Vittorio Colizzi

**Affiliations:** 1 Department of Experimental Medicine, University of Rome Tor Vergata, 00133 Rome, Italy; alessandro.terrinoni@uniroma2.it (A.T.); dimitri.koroliouk@ukr.net (D.K.); 2 Laboratoire des Grandes Epidemies Tropicales, Faculty of Medicine, University Hospital Complex “Bon Samaritain”, N’Djamena P.O. Box 456, Chad; dafogodjibagaouabel@gmail.com (A.D.D.); colizzi@uniroma2.it (V.C.)

**Keywords:** milk exosomes, microRNA targetome, in silico analysis, cross-species comparison, miR-148a, food–host RNA interactions, animal milk

## Abstract

Milk-derived extracellular vesicles (EVs) transport microRNAs (miRNAs) that are unusually stable and have been proposed to survive digestion and modulate gene expression in the consumer, although their dietary bioavailability and physiological relevance remain debated. How the predicted regulatory potential of these miRNAs differs among the milks of different animals most relevant to human nutrition has not been systematically compared. Here, we performed an integrative in silico analysis of publicly available small-RNA sequencing data from 29 milk and milk-cell samples of human, cow, goat, and donkey origin. miRNAs were quantified against human (hsa) miRBase references—thereby restricting the analysis to evolutionarily conserved miRNAs with human orthologs—and their predicted effect on the human transcriptome was modeled by integrating predicted (mirDIP database) and experimentally supported (TarBase v9 database) miRNA–target interactions into a per-gene, per-species weighted targeting score. Because miRNAs act predominantly as repressors, this score is read as a prediction of which genes would be post-transcriptionally down-regulated in a recipient. miR-148a-3p dominated the exosomal spectrum of all four species (human, cow, goat, and donkey; ≈21.5% of pooled abundance), and the twenty most abundant miRNAs accounted for roughly three quarters of the signal. Of 4577 robustly targeted genes, a 1809-gene conserved “pan-milk” core showed the highest cross-species targeting and was enriched for transcriptional regulation, PI3K–Akt, MAPK, and TGF-β/SMAD signaling, autophagy and—strikingly—the components of the RNA-interference machinery itself. Species-restricted gene sets recapitulated biologically plausible programs, including a human-biased neuronal/axon-guidance and chromatin module, a donkey-biased transcriptional, epithelial, and immune (*CD47*) module, and a ruminant lipid/cholesterol and insulin–mTOR module. Across categories, we observed a reproducible confidence–exclusivity trade-off. We emphasize that these results are computational predictions that assume dietary miRNA uptake and do not constitute experimental validation. We provide the complete targetome as a hypothesis-generating resource to prioritize candidate genes, pathways, and milk types for future functional, nutritional, and epigenetic investigation. Across the 29 samples from the four species, miRNA composition segregated by species (silhouette width = 0.82, a cluster-separation measure ranging from −1 to 1, with values near 1 indicating well-separated groups) and the category structure exceeded a permutation null, indicating that the between-species signal is robust to differences in dataset origin and milk state.

## 1. Introduction

Milk is increasingly recognized as a complex signaling fluid rather than a purely nutritional secretion, providing the neonate with bioactive proteins, oligosaccharides, hormones, and nucleic acids in addition to macronutrients; breastfeeding is associated with broad and durable benefits for infant and maternal health [1]. A substantial fraction of milk’s non-nutritional bioactivity is carried by extracellular vesicles (EVs), including exosomes, which were first shown to shuttle functional mRNA and microRNA between cells by Valadi and colleagues [2]. Exosomes with immunomodulatory features are abundant in human breast milk [3], and milk was subsequently established as one of the richest known biological sources of microRNAs (miRNAs), with immune-related species particularly enriched in the exosomal fraction [4,5]. Because the operational definition and purification of EV subpopulations remain methodologically heterogeneous, we use the terms “EV” and “exosome” in the inclusive sense recommended by the International Society for Extracellular Vesicles, recognizing that the small-RNA cargo analyzed here is derived from mixed EV preparations [6].

MicroRNAs are short non-coding RNAs that guide post-transcriptional repression of target mRNAs through seed-region complementarity, predominantly within 3′ untranslated regions [7]. Conserved seed pairing implies that a large proportion of protein-coding genes are under selective pressure to maintain miRNA target sites, such that individual miRNAs can each regulate hundreds of transcripts and most mammalian mRNAs are conserved targets [8,9]. Importantly, the canonical consequence of this targeting is repression—typically a modest, fine-tuning reduction in target protein output rather than an on/off switch [10,11]—so a map of the genes most strongly targeted by milk miRNAs is, in effect, a prediction of which transcripts would be post-transcriptionally down-regulated in a recipient cell. Deeply conserved families such as let-7 exemplify the ancient and pleiotropic nature of this regulation [12]. The hypothesis that dietary miRNAs might act analogously in a consumer organism gained prominence after the report that a plant miRNA, MIR168a, could be detected in mammalian serum and influence a mammalian transcript, suggesting cross-kingdom regulation [13].

Whether mammalian dietary miRNAs are absorbed in functionally meaningful amounts is, however, genuinely contested, and we treat this debate as central to the interpretation of our work. Several groups have reported absorption of bovine-milk miRNAs and exosomes and downstream effects on gene expression, as well as endocytic uptake of milk exosomes by intestinal, endothelial, and immune cells [14,15,16,17]. Mechanistically, milk-derived miRNAs resist simulated gastrointestinal digestion [18], orally administered milk exosomes distribute to defined tissues with cargo-specific patterns [19], and a stomach SID-1 transmembrane family member (*SIDT1*) has been proposed as a route for dietary miRNA uptake [20]. Other studies, however, have found little evidence for biologically relevant uptake of dietary miRNAs in adults, or have attributed apparent signals to endogenous synthesis and detection artifacts [21,22,23]. Comprehensive reviews conclude that, while milk EVs and their cargo possess bioactivity in model systems, the quantitative contribution of orally ingested miRNAs to host gene regulation in vivo is not resolved [24,25]. Accordingly, the present study deliberately adopts uptake as a working premise in order to model what regulatory landscape would be engaged if milk exosomal miRNAs reach recipient cells; it does not, and cannot, demonstrate that such regulation occurs in vivo.

This modeling perspective is motivated by the milk epigenetic-programming hypothesis, in which milk miRNAs—most prominently miR-148a-3p, a direct repressor of the maintenance DNA methyltransferase *DNMT1*—are proposed to participate in the epigenetic and metabolic programming of the developing infant [26,27]. Milk-derived miRNAs are notably resistant to degradation owing to vesicle encapsulation [28], which is the property that makes a dietary-exposure scenario, particularly through raw or minimally processed milk, biologically conceivable. Milk composition and its EV miRNA cargo differ substantially among species [29], and these differences have practical relevance: donkey milk, for example, is widely studied as a hypoallergenic substitute and is tolerated by a majority of children with cow’s milk protein allergy [30,31]. If the regulatory potential of milk miRNAs is real, then the choice of milk species could, in principle, bias which host pathways are most strongly engaged.

Comparative work has begun to contrast milk extracellular-vesicle RNA across livestock species—most directly, the cow-, donkey-, and goat milk-EV transcriptomes profiled by Mecocci et al. [29]—but these efforts have catalogued the miRNAs themselves rather than their integrated regulatory potential, have not placed human milk alongside livestock milks, and have not weighted predicted targeting by miRNA abundance or cross-referenced it against experimentally supported interactions. To our knowledge, an abundance-weighted, multi-evidence targetome of the human transcriptome that situates human, cow, goat, and donkey milk within a single comparative framework has not previously been reported.

We addressed this gap by quantifying the exosomal miRNA spectra of human, cow, goat, and donkey milk and mapping them to a common human reference. Mapping to human (hsa) miRBase annotations is a deliberate choice with two consequences: it focuses the analysis on the conserved miRNAs that possess human orthologs—precisely the population most plausibly capable of acting on the human transcriptome—while necessarily excluding lineage-specific miRNAs that lack a human counterpart. We consider this an appropriate framing for the question of effect on a human consumer, and we discuss its limitations explicitly in Section 3.6.

The aims of this study were therefore threefold: (i) to characterize and compare the exosomal miRNA abundance spectra of the four milks; (ii) to construct an abundance-weighted, cross-species map of predicted regulatory pressure on the human transcriptome, integrating both predicted and experimentally supported interactions; and (iii) to identify a conserved regulatory core shared by all milks and the species-specific signatures that distinguish them, and to interpret these in the light of known biology while remaining within the bounds of an in silico prediction. We provide the full underlying data as Supplementary Materials (S1–S11: per-species and per-sample miRNA abundance spectra, pooled cross-milk rankings, the categorized gene targetome and its target tables, robustness analyses, sample provenance, and the miRNA–gene edge list) and frame the entire analysis as hypothesis-generating: a structured prioritization of candidates for the experimental, nutritional, and epigenetic studies that would be required to test them.

## 2. Results

### 2.1. The Milk Exosomal microRNA Landscape Is Dominated by a Small, Shared Set of Conserved miRNAs

We assembled small-RNA sequencing data from 29 milk and milk-cell samples spanning four species: human (*n* = 8), cow (*n* = 13), goat (*n* = 4), and donkey (*n* = 4), drawn from independent public studies to mitigate single-cohort bias [5,29,32,33,34] (accessions are listed in Section 4 and the Data Availability Statement). Reads were processed and quantified against human miRBase references using an established miRNA discovery and quantification workflow [35,36], yielding 1549 distinct conserved miRNAs across the dataset, of which 456 were detected in all four species. The per-species relative abundance of every detected miRNA is provided in Supplementary Material S1, and the complete per-sample matrix across all milks is provided in Supplementary Material S2.

**Figure 1:**
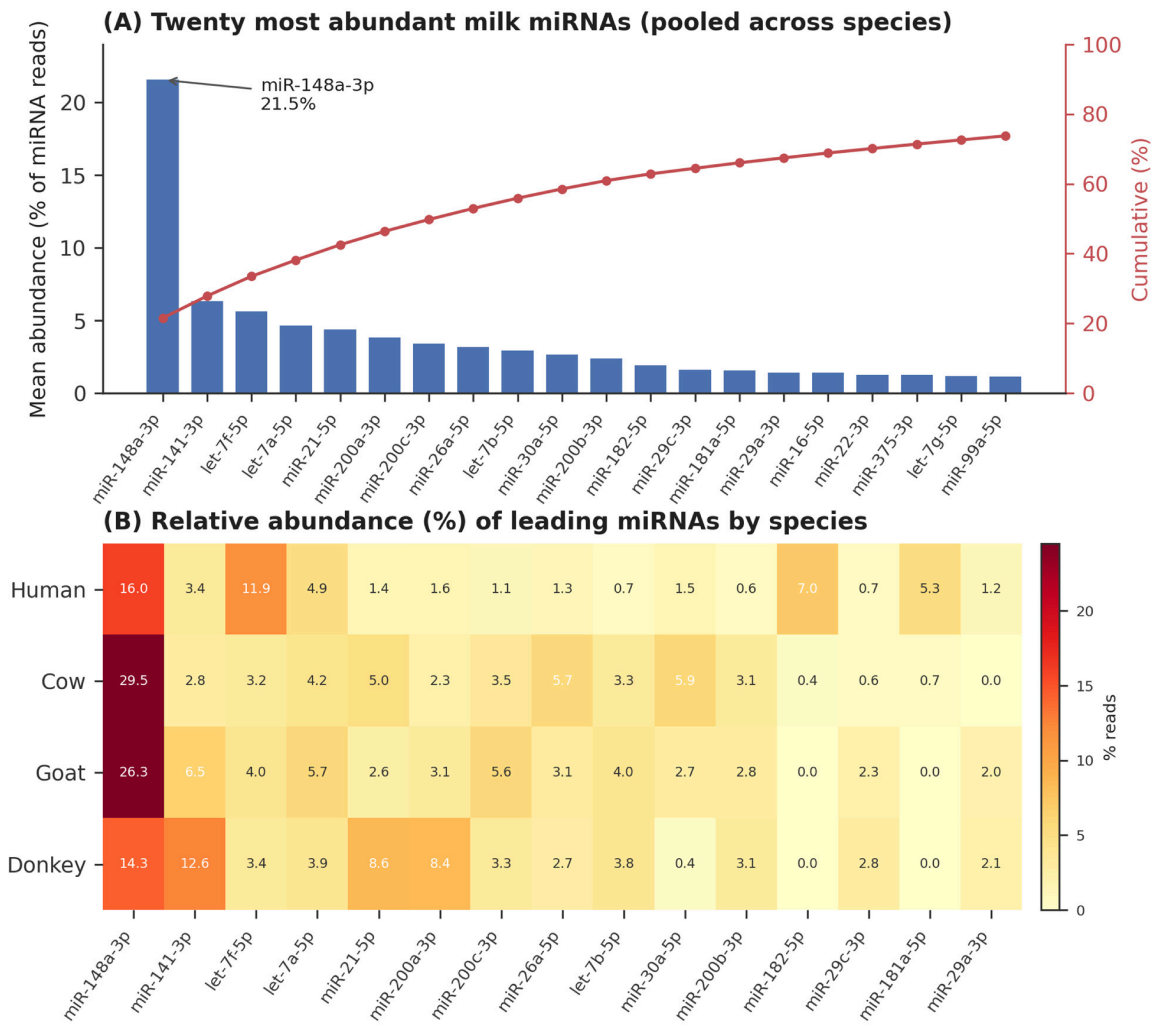
The milk exosomal microRNA landscape. (**A**) The twenty most abundant pooled miRNAs (bars, left axis) and the cumulative fraction of total abundance they represent (line, right axis); miR-148a-3p alone contributes ≈21.5% and the top twenty contribute ≈74%. (**B**) Heatmap of the relative abundance of the fifteen most abundant miRNAs across human, cow, goat, and donkey milk, showing a broadly conserved compositional structure. Values are derived from the per-species and pooled abundance tables in Supplementary Materials S1–S3. Because miRNAs were quantified against human references, the spectra represent conserved miRNAs only.

The exosomal spectrum was strongly skewed toward a few highly abundant species. Pooling relative abundances across milks (Supplementary Material S3), miR-148a-3p—a conserved, milk-enriched miRNA that represses the maintenance DNA methyltransferase DNMT1 and is implicated in DNA-methylation, immune, and metabolic regulation [28]—was the single most abundant miRNA in every species, contributing on average ≈ 21.5% of the total signal. The five most abundant miRNAs together accounted for ≈42.5% of abundance, the top ten accounted for ≈58.5%, the top twenty accounted for ≈73.7%, and the top fifty accounted for ≈90.5% (Figure 1A). The dominant miRNAs—including miR-148a, miR-141, members of the let-7 family (let-7a/b/f/g/i), miR-21, the miR-200 family (miR-200a/b/c), miR-26a, miR-30a/d, miR-182, miR-29a/c, miR-181a, miR-22, miR-375, and miR-99a—are all previously documented milk-EV miRNAs, supporting the biological credibility of the spectra. The relative composition of the fifteen most abundant miRNAs was broadly conserved across species, with quantitative rather than qualitative differences (Figure 1B); the near-universal presence and high rank of miR-148a and let-7 members is consistent with their reported role as obligate, abundant constituents of well-prepared milk small-RNA libraries.

### 2.2. An Abundance-Weighted, Cross-Species Targetome of the Human Transcriptome

To translate these abundance spectra into predicted regulatory pressure, we constructed a weighted targeting score for each human gene in each species. For every detected miRNA, we retrieved predicted human targets from mirDIP, which integrates multiple prediction algorithms into a unified confidence ranking [37,38], and experimentally supported interactions from TarBase v9, which catalogues miRNA–gene interactions backed by published experiments [39]. For each gene–species pair, contributions from individual miRNAs were weighted by the relative exosomal abundance of those miRNAs in that species and combined with their interaction confidence to produce a continuous targeting score (spanning approximately 0–100 in this dataset). Because miRNAs act predominantly as repressors [7,10,11], a higher score denotes a stronger prediction that the gene would be post-transcriptionally down-regulated when the targeting miRNAs are present; as discussed below, this directional reading is clear at the level of individual genes but not, by itself, at the level of whole pathways. As the user-defined analytical design intended, the weighting reflects primarily the presence and relative abundance of targeting miRNAs rather than absolute molecular stoichiometry; the number of supporting mirDIP predictions and TarBase experiments was additionally retained as separate columns to aid interpretation. The complete weighted targetome—comprising every retained gene with its four per-species scores, fold-change metric, assigned category, and prediction/experimental support counts—is provided as Supplementary Material S4.

To focus on robustly targeted genes and remove background noise, genes whose summed cross-species score fell below 10 (a minimal aggregate-evidence cut-off), or whose maximum single-species score fell below an empirical noise floor T (the 60th percentile of all non-zero targeting scores), were filtered out, removing 6039 weakly or non-specifically targeted genes and retaining 4577 genes for downstream analysis. Each retained gene was then assigned to a category by ranking its four per-species scores in descending order and identifying the dominant species (or group) as those lying above the first ≥1.5-fold drop between consecutively ranked scores; genes with no such gap were labeled Core_Pan_Milk when all four scores remained high (at or above the noise floor T) and Variable_Gradient otherwise. For reporting, a fold change (FC) was computed for each gene as the mean score of its dominant group divided by the highest-scoring non-dominant species, providing a measure of how exclusively a gene is targeted by a particular milk. We stress that this categorization is intended for enrichment analysis and for the detection of pronounced cross-species deviations rather than as a claim that a gene is targeted only by its assigned category; a gene assigned to one category may still be targeted by other milks, but is expected to be most strongly influenced by the milk type(s) defining its category.

### 2.3. A Conserved Pan-Milk Regulatory Core

**Figure 2:**
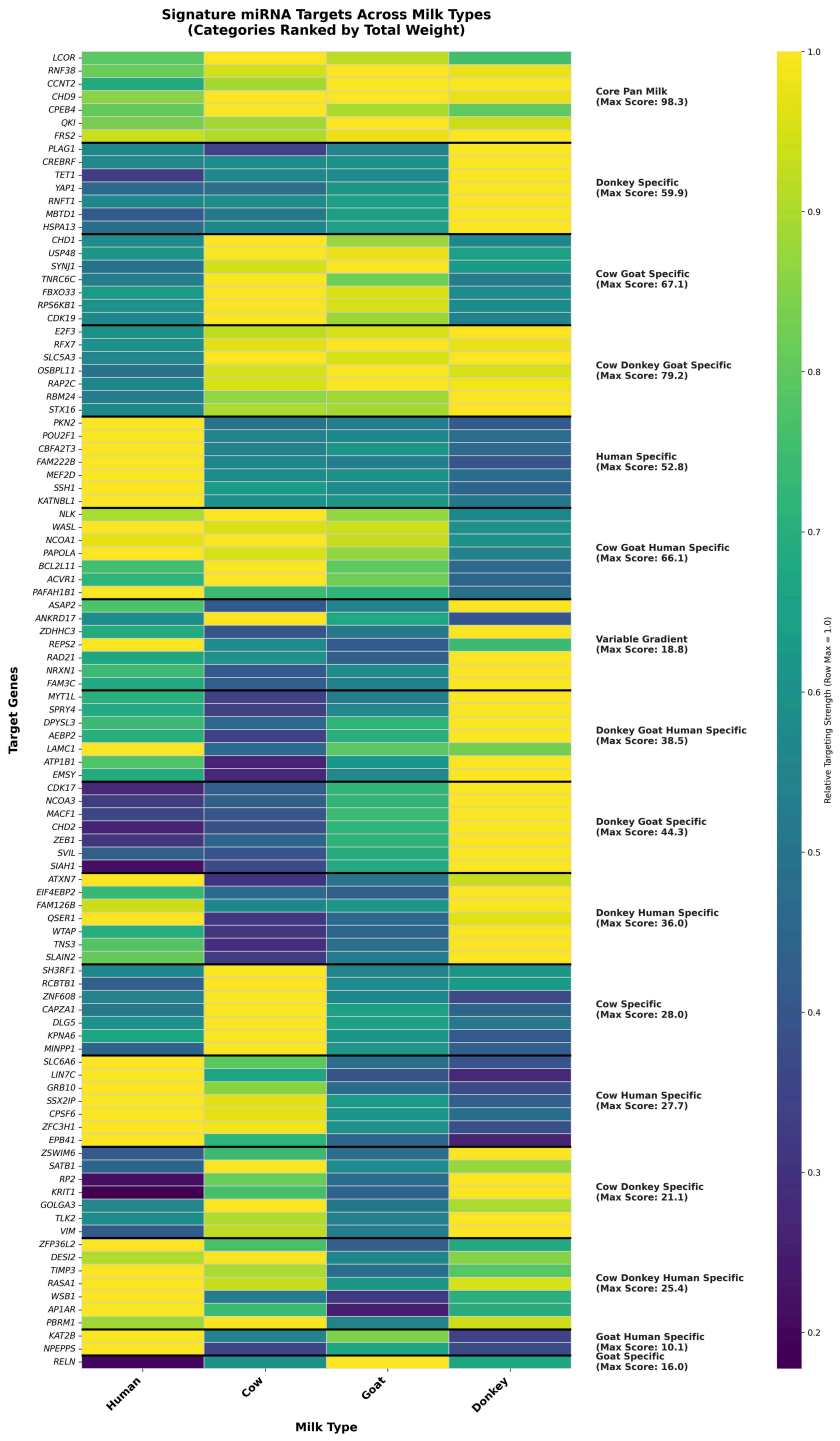
Representative signature genes across milk types for every category, ordered by total category weight. Each row is a gene and each column a milk; color encodes the relative targeting strength normalized within each gene (row maximum = 1.0), so it shows the cross-species pattern of a gene rather than its absolute score. Absolute magnitudes differ markedly between categories—indicated by the per-category maximum scores at right (for example, Core_Pan_Milk 98.3 versus Goat_Specific 16.0)—consistent with the confidence–exclusivity trade-off (Figure 3B). The conserved core (top block) is targeted comparably by all four milks, whereas the species-specific and multi-species blocks are brightest in their defining milk(s). All values are computational predictions; complete per-category gene lists are provided in Supplementary Material S5.

The largest category, Core_Pan_Milk, comprised 1809 genes (39.5% of the retained set) that were strongly and comparably targeted by all four milks (Table 1). This core had by far the highest mean dominant targeting score (74.8), reflecting balanced, high-confidence targeting across species (top block, Figure 2). Representative core genes and their per-species scores are listed in Table 2. Many core genes are central post-transcriptional and signaling regulators, including the transcriptional co-regulators *LCOR*, *CHD9*, and *CCNT2*; the RNA-binding and translational-control proteins *CPEB3*, *CPEB4*, *QKI*, and *NUFIP2*; the ubiquitin-system genes *RNF38* and *USP47*; the calcium-handling gene ATP2A2; and signaling hubs such as *PTEN*, *FRS2*, *TGFBR1*, and *SMAD2*. The high and even targeting of these genes across all milks identifies them as candidate constituents of a pan-mammalian milk regulatory program.

**Figure 3:**
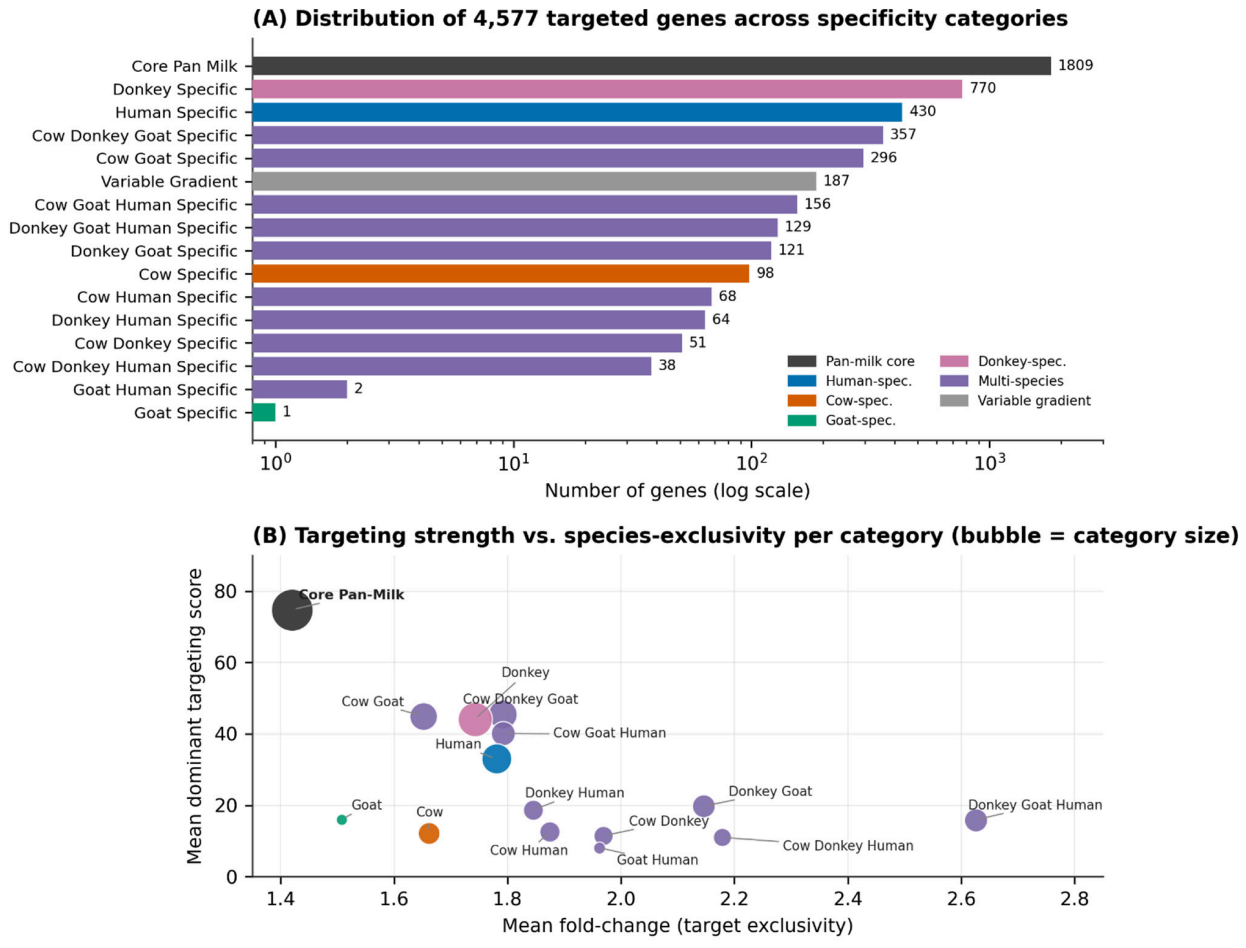
Architecture of the cross-species targetome. (**A**) The sixteen categories ranked by gene count (log scale); the Core_Pan_Milk category (1809 genes), the Donkey_Specific (770), and Human_Specific (430) categories are the largest. (**B**) Mean dominant targeting score versus mean fold change for each category (bubble area proportional to gene count), illustrating the confidence–exclusivity trade-off: more species-restricted categories show higher fold changes but lower absolute targeting scores. Category membership and statistics were derived from Supplementary Materials S4 and S5. In (**B**), bubble color denotes the category type: pan-milk core (dark grey), single-species signatures (human, blue; cow, orange; goat, green; donkey, pink), multi-species signatures (purple) and variable gradient (grey); bubble size is proportional to the number of genes in the category.

**Figure 4:**
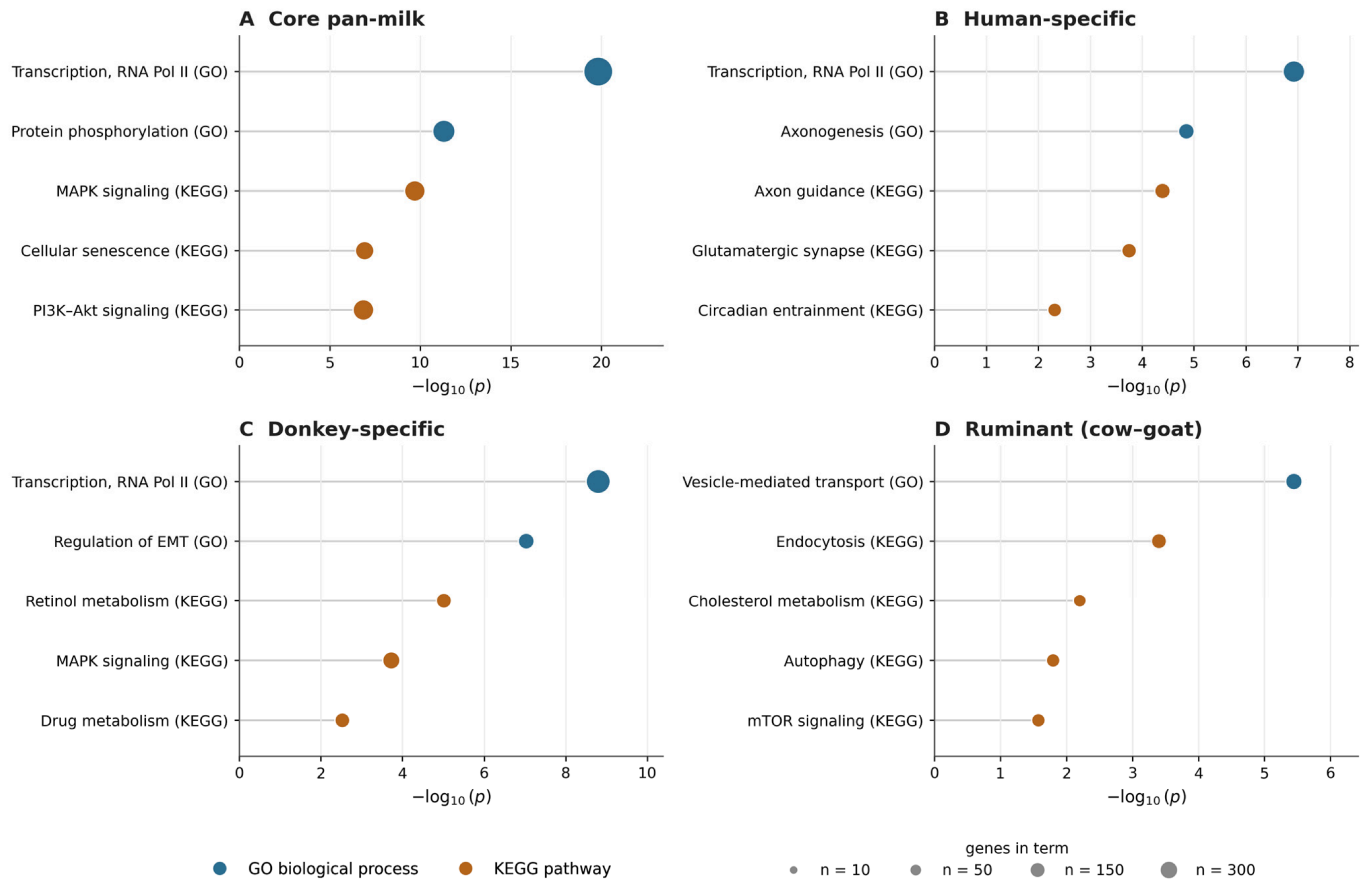
Weighted functional enrichment by category. Representative GO biological-process (blue) and KEGG pathway (orange) terms for the conserved Core_Pan_Milk set (**A**), the Human_Specific set (**B**), the Donkey_Specific set (**C**), and the ruminant Cow_Goat_Specific set (**D**). The x-axis is the enrichment significance (−log_10_*p*) and the marker area is proportional to the number of category genes annotated to the term (*n*). Terms were selected to represent the predominant predicted programs of each category; complete weighted-enrichment plots for all categories and for each milk globally are provided in Supplementary Materials S7 and S8. Because miRNAs repress their targets, enrichment identifies processes under predicted repressive (down-regulatory) targeting; as some targets are themselves regulators, term-level enrichment does not by itself indicate whether net pathway output rises or falls. All enrichment results are computational predictions and are not experimentally validated. Abbreviations: GO, Gene Ontology; KEGG, Kyoto Encyclopedia of Genes and Genomes; CRI, cumulative regulatory impact.

Functional enrichment of the core (Supplementary Material S7) was dominated, as expected for any broad miRNA targetome [7], by RNA polymerase II–dependent transcriptional regulation and by canonical growth and survival signaling, including the PI3K–Akt, MAPK, and TGF-β/SMAD pathways and macroautophagy [40,41]; the most strongly weighted KEGG terms additionally included pathways-in-cancer, cellular senescence, and the signaling pathways regulating pluripotency of stem cells (Figure 4A). A more distinctive observation was that the conserved core was itself enriched for the effectors of the RNA-interference machinery: the Argonaute proteins *AGO1* and *AGO4* (with *AGO2* appearing in a closely related multi-species category; Section 2.9), the GW182 paralog *TNRC6B*, and the regulatory RNA-binding proteins *CPEB3*/4, *QKI* and *NUFIP2*.

We interpret this cautiously as an intriguing hypothesis rather than an established mechanism: if exosomal miRNAs were taken up, their most conserved predicted targets would include the components that the recipient cell uses to execute miRNA-mediated silencing, raising the testable possibility of a homeostatic feedback in which incoming milk miRNAs, by repressing these effectors, would down-tune the recipient’s own silencing apparatus—the very machinery that would otherwise execute their effects. This self-referential targeting is presented strictly as a direction for future experimental work.

### 2.4. Category Structure Reveals a Confidence–Exclusivity Trade-Off

The 4577 retained genes partitioned into sixteen categories whose sizes spanned three orders of magnitude (Table 1, Figure 3A). After the conserved core, the next-largest categories were Donkey_Specific (770 genes), Human_Specific (430), the ruminant-plus-donkey Cow_Donkey_Goat_Specific group (357), and the ruminant Cow_Goat_Specific group (296). Single-species ruminant categories were comparatively small (Cow_Specific, 98; Goat_Specific, 1), and several two- and three-species combinations occupied intermediate sizes (Table 1). Representative signature genes for each category, together with their cross-species targeting patterns, are shown in Figure 2.

Plotting each category’s mean dominant targeting score against its mean fold change revealed a consistent trade-off (Figure 3B): as categories became more species-restricted, their mean dominant score declined while their mean fold change rose. The conserved core sat at high score and low exclusivity, whereas narrow multi-species categories such as Donkey_Goat_Human_Specific reached the highest mean fold changes (up to ≈2.6) at modest absolute scores. This pattern has both a statistical and a biological reading. Statistically, genes targeted by all milks accumulate high scores from many shared, abundant miRNAs, whereas genes whose targeting depends on a few lineage-biased miRNAs achieve high exclusivity but lower absolute weight. Biologically, it implies that the most species-discriminating regulatory signals are carried by lower-abundance, more divergent miRNAs and should be interpreted as candidate differentiators rather than dominant effects. We further note that the Goat_Specific category contained only a single gene. This near-absence has a largely structural explanation rather than implying that goat milk lacks regulatory targeting: the cow and goat miRNomes are closely related, so most goat targeting is shared with cow and is captured by the sizeable Cow_Goat_Specific category (*n* = 296). Because a gene is labeled goat-exclusive only when its goat score exceeds every other species—including cow—by the 1.5-fold gap, and goat and cow scores are typically similar, such genes are rare by construction. The smaller goat sample (*n* = 4) compounds this effect but is secondary to it, and we accordingly treat goat-exclusive signals with caution (Section 3.6).

### 2.5. Weighted Functional Enrichment Distinguishes Conserved from Divergent Programs

Weighted GO and KEGG enrichment of the categorized gene sets (Supplementary Material S7), and of each milk analyzed globally (Supplementary Material S8), made the conserved-versus-divergent contrast explicit (Figure 4). When milks were analyzed globally, all four species converged on the same dominant terms—regulation of transcription and the PI3K–Akt, MAPK, and TGF-β/SMAD axes, with “pathways in cancer” as the top KEGG category in every species (Supplementary Material S8). This convergence confirms that the broad signal is shared across milks and, as expected for large miRNA targetomes, is partly a generic property of abundant miRNA targeting rather than a milk-specific effect [7]. The category-restricted sets, in contrast, resolved distinct and biologically coherent programs (Figure 4): a transcription- and growth-signaling core (Figure 4A), a human-biased neuronal and synaptic module (Figure 4B), a donkey-biased transcriptional/epithelial, retinol-, and drug-metabolism module (Figure 4C), and a ruminant lipid/cholesterol and insulin–mTOR/autophagy module (Figure 4D). These category-level programs, which we describe in turn below, rather than the shared global signal, are where milk-type differences in predicted regulatory potential are concentrated.

A directional caveat applies throughout. Because miRNA targeting represses its targets, the genes populating each category are predicted to be post-transcriptionally down-regulated when the corresponding milk miRNAs are active, and the enriched terms in Figure 4 therefore identify processes placed under predicted repressive pressure. This direction is unambiguous for individual target genes but not for whole pathways, because many targets are themselves regulators and repression of a negative regulator de-represses its own downstream effectors. Within the conserved core, for instance, *PTEN*—an inhibitor of PI3K–Akt—is a high-scoring target, so its predicted repression would relieve rather than suppress that pathway, whereas predicted repression of the positive transducers *TGFBR1* and *SMAD2* would instead dampen TGF-β/SMAD output, and repression of transcriptional corepressors such as *LCOR* would de-repress their target genes. We therefore interpret directionality strictly at the gene level (these genes are predicted to be down-regulated) and read pathway-level enrichment as identifying processes under regulatory pressure, without inferring whether net pathway activity rises or falls.

### 2.6. A Human-Specific Signature Enriched for Neuronal and Chromatin Programs

Among the 430 Human_Specific genes (Table 3; Figure 4B), the sharpest human-exclusive signal was NR4A3, an immediate-early nuclear receptor, which combined a high fold change (FC = 5.44) with predicted human-biased targeting. Additional human-biased targets included the transcription factors POU2F1, MEF2D, and CREB1 and the metabotropic glutamate receptor GRM5 (FC = 2.42). Functional enrichment of the human-specific set (Supplementary Materials S7 and S8) highlighted axonogenesis and axon guidance, the glutamatergic synapse, RNA polymerase II transcription, and chromatin remodeling. Because miRNA targeting represses these genes, the predicted effect would be to restrain or fine-tune—rather than promote—axon-guidance and synaptic programs; such damping is, if anything, directionally compatible with the protracted, altricial trajectory of human neurodevelopment, in which prolonged postnatal maturation is characteristic. We stress that this is only a hypothesis consistent with known human developmental biology, not evidence that human milk miRNAs either drive or retard neuronal programs.

### 2.7. A Donkey-Specific Signature Enriched for Transcriptional, Epithelial, and Immune Targets

Donkey milk produced the second-largest category (770 Donkey_Specific genes; Table 3; Figure 4C). Prominent donkey-biased targets included the developmental transcription factors *PLAG1* and *YAP1*, the DNA demethylase *TET1*, the large E3 ligase *HECTD1* (notably high experimental support, 1405 TarBase experiments), and—of particular interest—*CD47*, the “don’t-eat-me” signal that restrains phagocytic clearance, which showed a high dominant score (46.9; the abundance-weighted targeting-strength measure defined in Section 4.5) and an FC of 2.34. Enrichment of the donkey set pointed to transcriptional and epithelial-to-mesenchymal regulation, retinol and xenobiotic metabolism, and immune regulation. This predicted transcriptional and immune-gene bias is broadly concordant with the established clinical observation that donkey milk is well tolerated and frequently used as a hypoallergenic substitute [30,31], and with reports of anti-inflammatory and immunomodulatory properties of donkey-milk EVs [29]. Here, the directional caveat is especially important: because targeting represses its target, predicted down-regulation of *CD47* would lower a “don’t-eat-me” brake rather than reinforce immune tolerance, so the net immunological consequence cannot be read off the targeting direction alone. We therefore present these as gene-level predictions and concordances worth testing, not as a demonstrated immunomodulatory mechanism.

### 2.8. A Ruminant Lipid and Insulin–mTOR Module

Genes shared between the two ruminants (Cow_Goat_Specific, 296 genes; Table 3; Figure 4D) were enriched for lipid and cholesterol metabolism and for insulin–mTOR signaling, with representative targets including the LDL receptor *LDLR*, the insulin-receptor substrate *IRS1*, and the mTOR effector ribosomal protein S6 kinase RPS6KB1, alongside the deubiquitinase *USP48* and the chromatin remodeler *CHD1*. Because these are positive effectors of cholesterol uptake and growth signaling, their predicted repression points to a coherent down-regulatory (restraining) tone on lipid handling and insulin–mTOR growth signaling—one of the clearest directional readings in the dataset. The cow-only set (Cow_Specific) added transcriptional and chromatin regulators such as NR4A2, the Polycomb methyltransferase *EZH2*, and the *ATF1*/*ATF2* factors. The predicted ruminant emphasis on lipid handling and growth-coupled insulin–mTOR signaling is consistent with the metabolic and lactational physiology of dairy ruminants, but, as elsewhere, breed, diet, and lactation stage are known to influence milk miRNA content and would need to be controlled in any confirmatory study.

### 2.9. Convergent Multi-Species Modules and Notable Individual Targets

Several multi-species categories captured biologically coherent modules (Table 3). The Donkey_Goat_Specific set was enriched for epithelial-barrier and cytoskeletal regulators, including the adherens-junction protein *CTNND1* (the highest-FC gene in this group at 4.01), the cytoskeletal cross-linker *MACF1*, the small GTPase *CDC42*, the EMT factor *ZEB1*, and the angiogenic factor *VEGFA*. The Donkey_Goat_Human_Specific set—which excludes cow—was distinguished by a structurally striking observation: a cluster of fourteen α-protocadherin genes (*PCDHA1*–11 and the PCDHA-C variants), all with fold changes near 3.2, were predicted to be targeted by all milks except bovine. Because these genes are physically clustered and functionally linked to neuronal identity, their coordinated, bovine-excluded targeting is, to our knowledge, a novel observation that merits dedicated investigation, while remaining a prediction. The Cow_Human_Specific set contained the imprinted IGF-pathway adaptor *GRB10* and the taurine transporter SLC6A6; the Donkey_Human_Specific set contained the m6A-methyltransferase component *WTAP*, the lipid-sensing receptor *PPARA*, and the longevity-associated *FOXO3* and EIF4EBP2.

Finally, two individual targets are noteworthy for their support metrics rather than their exclusivity. *AGO2*, the catalytic core of the RNA-induced silencing complex, carried the highest experimental support of any gene in the dataset (1940 TarBase experiments) and fell in the Cow_Donkey_Human_Specific category together with chromatin readers such as *PBRM1*, *BRD1*, and *NSD3*—reinforcing the self-targeting-of-the-silencing-machinery hypothesis noted for the core (Section 2.3). The highest fold change in the entire dataset belonged to the zinc-finger factor *ZBTB43* (5.79; Donkey_Goat_Human_Specific). Representative signature genes for each major category, with their dominant scores, fold changes, and experimental support, are compiled in Table 3, and the complete top-50 gene lists per category are provided in Supplementary Material S5.

### 2.10. Robustness, Statistical Structure and Enrichment Caveats

**Figure 5:**
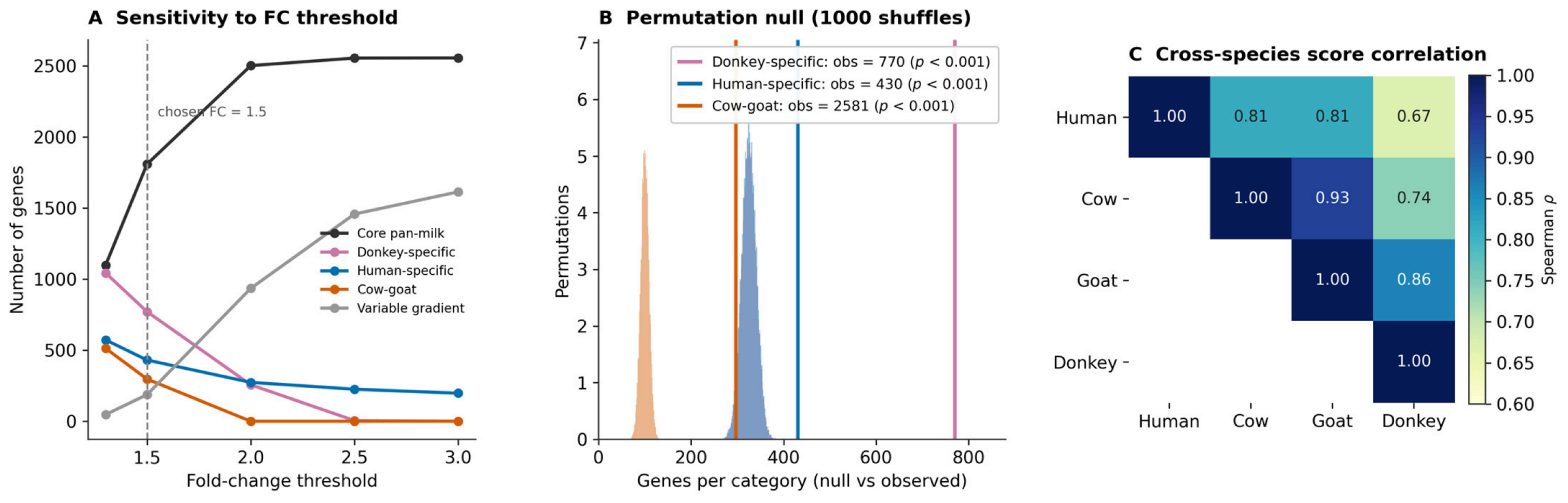
Robustness and statistical structure of the targetome. (**A**) Gene counts of the major categories as a function of the fold-change threshold; the retained-gene count is fixed by the noise filter and the chosen threshold (1.5, dashed line) lies within a stable regime. (**B**) Permutation null distributions (1000 within-gene score shuffles) for the three largest species-restricted categories; vertical lines mark the observed counts, which exceed the nulls in every case (empirical *p* < 0.001). (**C**) Spearman correlation between the per-species weighted-score vectors over the 4577 retained genes. All values are computational and descriptive.

We next examined how strongly the category architecture depends on the analysis parameters (Figure 5A). The number of retained genes (4577) is determined only by the noise filter and is independent of the fold-change threshold; varying the noise filter across the 50th–70th score percentile and a minimum summed score of 5–20 moved the retained set smoothly between 3353 and 5565 genes without changing the qualitative structure. The categorical architecture—a large conserved core with donkey- and human-biased sets as the largest species-restricted categories and a substantial cow–goat co-dominant set—was itself stable across fold-change thresholds from 1.3 to 2.0 and collapsed (specific categories folding into the core and the variable-gradient class) only at stringent thresholds of 2.5 or above. The chosen value of 1.5 thus lies within a stable regime rather than at a sensitive boundary.

To test whether the species attribution of targeting is more than a by-product of the assignment rule, we permuted the four per-species scores within each gene 1000 times and re-derived the categories (Figure 5B). Because this preserves each gene’s score profile, the total number of species-restricted genes is fixed by construction (2581); the permutation randomizes only which species receive the exclusive signal. The observed sizes of the major species-restricted sets greatly exceeded their permutation null (a randomization test in which category labels are reshuffled many times to build the distribution of category sizes expected by chance)s—Donkey_Specific 770 versus 325 ± 16, Human_Specific 430 versus 325 ± 16, and Cow_Goat_Specific 296 versus 100 ± 9 (empirical *p* < 0.001 in every case)—indicating that the species and co-dominance patterns reflect genuine structure rather than random assignment. Consistent with this, the per-species score vectors were all positively correlated but clearly tiered (Figure 5C): cow and goat were the most similar (Spearman ρ = 0.93), whereas donkey was the most distinct from human (ρ = 0.67). The high cow–goat correlation gives a quantitative basis for the near-absence of goat-exclusive genes (Section 2.4)—goat targeting is largely shared with cow and is absorbed into the cow–goat category—and the uniformly positive correlations indicate that, despite differing alignment rates, the per-species relative-abundance-weighted scores are mutually consistent and dominated by the shared core, supporting cross-species comparison.

A statistical caveat applies specifically to the functional-enrichment results. Standard over-representation tests are biased for miRNA target sets, because predicted miRNA targets are enriched for genes with long 3′ untranslated regions and many target sites, so that even randomly chosen miRNAs can yield apparently significant enrichment [42]. Our data show this directly: conserved-core genes are targeted by far more miRNAs than species-specific genes (mean 15.3 versus 8.1 distinct mirDIP miRNAs), and targeting breadth correlates with the weighted score (Spearman ρ = 0.62). The broadly recurring core themes (transcriptional regulation, PI3K–Akt, and MAPK signaling) should therefore be read with this bias in mind rather than as strictly core-specific biology; the species-restricted contrasts, which compare miRNA-target sets against one another rather than against the whole genome, are less susceptible to this artifact and carry greater interpretive weight. The full threshold-sensitivity and permutation results are provided as Supplementary Material S9 and in the deposited analysis code.

### 2.11. Cross-Dataset Comparability of the Species Spectra

**Figure 6:**
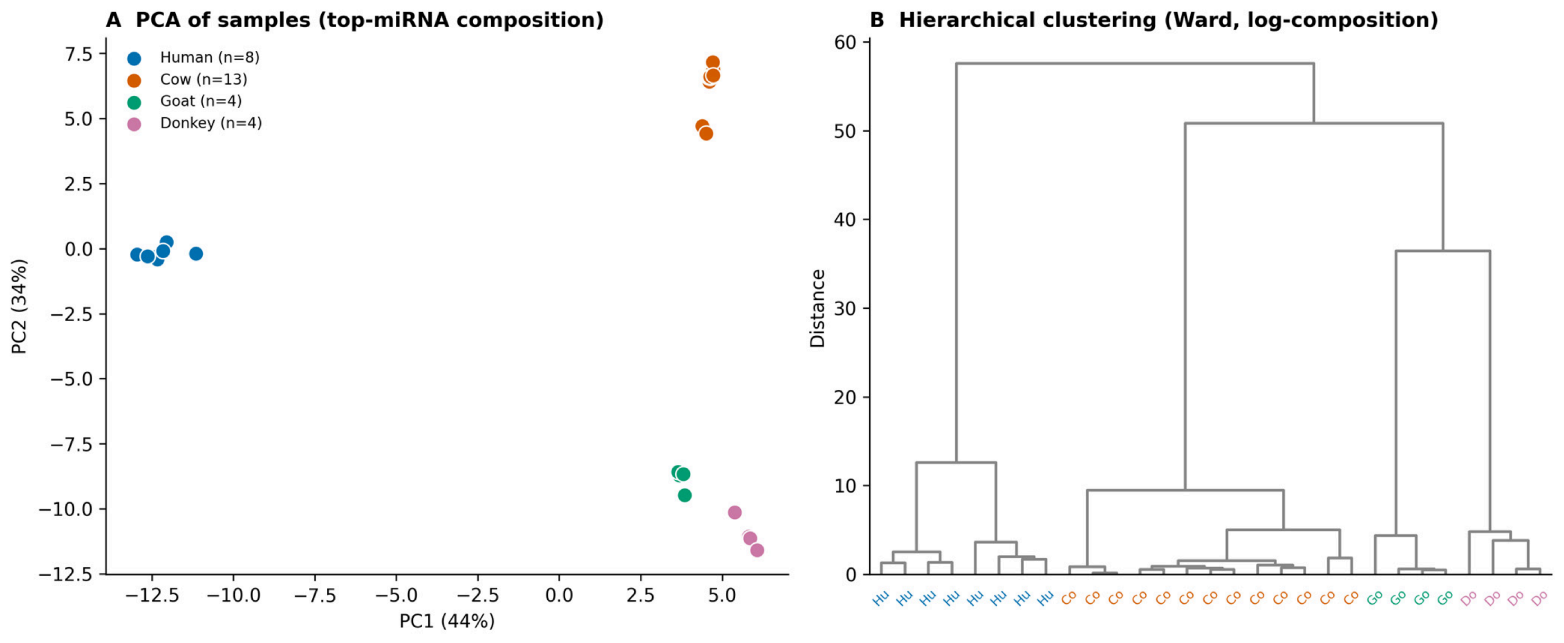
Samples group by species despite heterogeneous origins. (**A**) Principal-component analysis of the per-sample relative abundance of the conserved miRNAs (29 samples; color = species). (**B**) Ward hierarchical clustering of the same data; every sample joins its own species (leaf color = species). Mean silhouette width = 0.82; mean between-species distance 6.1-fold greater than within-species distance. Input datasets and their characteristics are listed in Supplementary Material S10. Species abbreviations: Hu, human; Co, cow; Go, goat; Do, donkey.

Because the samples were drawn from independent studies that differ in milk type (mature milk, colostrum, or milk fractions), isolation method, cohort, and platform (Supplementary Material S10), we asked whether these technical and biological differences, rather than species, might drive the observed structure. Unsupervised analysis of the per-sample miRNA composition argues against this: in the principle-component space, the 29 samples were separated cleanly by species (Figure 6A), and Ward hierarchical clustering grouped every sample with its own species (Figure 6B). The separation was strong—a mean silhouette width of 0.82 (the silhouette width is a clustering metric between −1 and 1, where higher values indicate that samples lie closer to their own species than to any other), with the mean between-species distance 6.1-fold greater than the within-species distance—indicating that the species identity of the milk, not dataset origin or milk state, is the dominant source of variation in the conserved-miRNA spectra. The analysis also recovers the expected biological relationships, with cow and goat most similar and donkey most distinct (Figure 5C). This is consistent with the permutation and correlation analyses above (Section 2.10) and supports the cross-species comparison, while residual effects of dataset heterogeneity are treated as a limitation (Section 3.6).

### 2.12. Conserved miRNAs Converge on Shared Core Target Genes

**Figure 7:**
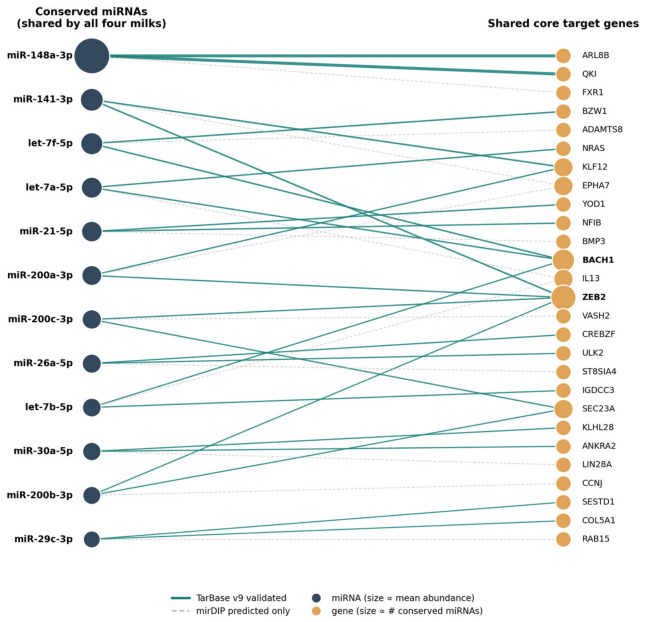
Conserved miRNAs converge on shared core target genes. Bipartite network of the twelve most abundant miRNAs present in all four milks (left; node size proportional to mean abundance) and their strongest predicted targets among Core_Pan_Milk genes (right; node size proportional to the number of conserved miRNAs targeting the gene). Teal solid edges are interactions documented in TarBase v9; grey dashed edges are mirDIP-predicted only. *ZEB2* and *BACH1* (bold) are the principal convergence hubs. The full edge list is provided in Supplementary Material S11.

To make the shared regulatory backbone explicit, we constructed a network linking the miRNAs detected in all four milks to their predicted targets within the conserved core (Figure 7). The eighteen pan-species miRNAs—led by miR-148a-3p, the let-7 family, miR-21-5p, and the miR-200 family—converge on a compact set of shared core genes, several of which are targeted by more than one conserved miRNA. The clearest convergence point is *ZEB2*, a predicted target of four of the pan-species miRNAs (the miR-200/miR-141 axis), followed by *BACH1* and *KLF12*. Because mirDIP predictions are made against the human transcriptome, these miRNA–target relationships are shared across species by construction; what the network adds is that the most abundant, universally present miRNAs concentrate their predicted repression on a small number of shared hubs and that the majority of the displayed core interactions (24 of 36) are additionally documented in TarBase v9. This convergence provides a concrete, experimentally anchored set of first candidates for the validation experiments outlined in Section 3.7.

## 3. Discussion

By integrating exosomal miRNA abundance with predicted and experimentally supported target interactions across four milks, this in silico study provides a structured comparison of the regulatory potential that human, cow, goat, and donkey milk could, in principle, exert on the human transcriptome. Three findings stand out: a large conserved core of genes targeted comparably by all milks; a set of species-specific and multi-species signatures that recapitulate biologically plausible programs; and a systematic confidence–exclusivity trade-off relating a category’s exclusivity to the abundance of the miRNAs that define it. We discuss each in turn, then address the central caveat of dietary bioavailability and the study’s limitations.

### 3.1. The Conserved Core as a Candidate Pan-Mammalian Milk Program

The conserved core was enriched for transcriptional regulation and for the PI3K–Akt, MAPK, and TGF-β/SMAD pathways [40,41], which together govern cell growth, differentiation, and survival. It is important to acknowledge that broad enrichment for transcriptional regulation and growth signaling is partly a generic property of any large miRNA targetome, because abundant miRNAs collectively target a substantial fraction of the transcriptome [7,8,9]. The biologically informative signal in our data is therefore not the presence of these pathways per se but the contrast between the balanced, high-confidence core and the divergent species-specific categories. Within that framing, the most distinctive feature of the core was its enrichment for the RNA-interference machinery itself—*AGO1*/*AGO4* (and *AGO2* in a closely related category), *TNRC6B*, and the RNA-binding regulators *CPEB3*/4, *QKI*, and *NUFIP2*.

We propose, as a hypothesis for experimental testing, that conserved milk miRNAs may preferentially engage the recipient’s silencing apparatus, which could provide a homeostatic buffering or recalibrating influence on endogenous miRNA activity. This idea is speculative and would require demonstration of uptake, target engagement, and functional consequence before it could be regarded as more than a prediction. We also asked whether the conserved core is inflated by highly connected “hub” genes, as expected from the known target-set bias of enrichment analysis [42]. Indeed, core genes are predicted targets of markedly more miRNAs than species-specific genes (mean 15.3 versus 8.1 distinct targeting miRNAs), and targeting breadth correlates with the weighted score (Figure 5; Spearman ρ = 0.62). We therefore treat the core’s generic functional themes with caution and place greater interpretive weight on the species-restricted contrasts, which compare miRNA-target sets against one another rather than against the whole genome.

### 3.2. miR-148a Dominance and the Epigenetic-Programming Hypothesis

The dominance of miR-148a-3p in all four species is consistent with prior milk miRNome studies and is mechanistically interesting because miR-148a directly represses *DNMT1*, the maintenance DNA methyltransferase [26]. This is a useful worked example of the framework’s directional logic: since the canonical action of a miRNA is to repress its target [7,10,11], abundant miR-148a predicts down-regulation of *DNMT1* and therefore a hypomethylating tendency, while the abundant let-7 family predicts down-regulation of proliferation regulators and thus a growth-restraining tendency [12]—a coherent, directionally explicit reading that does not depend on resolving the pathway-sign ambiguities discussed above. Within the milk epigenetic-programming framework, abundant miR-148a has been proposed to contribute to the epigenetic and metabolic shaping of the developing infant, potentially together with mTORC1-activating signals [27,43,44]. Our finding that miR-148a is the single most abundant exosomal miRNA in every species examined provides a quantitative substrate for this hypothesis and suggests that, if milk miRNAs are bioactive, a *DNMT1*-centered epigenetic axis would be engaged regardless of milk species, with species differences layered on top of this shared baseline. The conserved prominence of let-7 family members [12] similarly points to a shared, deeply conserved regulatory backbone.

### 3.3. Species-Specific Signatures as Candidate Adaptations

The species-specific categories were notable for their concordance with known physiology. The human-specific bias toward neuronal, axon-guidance, and chromatin programs aligns with the protracted, altricial neurodevelopment of human infants; the donkey-specific bias toward transcriptional, epithelial, and immune targets—including the tolerogenic *CD47* signal—aligns with the documented hypoallergenicity and immunomodulatory profile of donkey milk [29,30,31,45]; and the ruminant lipid/insulin–mTOR module aligns with dairy-ruminant metabolic physiology. The coordinated, bovine-excluded targeting of the clustered α-protocadherin genes is, to our knowledge, a novel observation that may reflect lineage-specific differences in the miRNAs that target this neuronal-identity locus. We are deliberately cautious about these interpretations: concordance with physiology is not causation, and we explicitly avoid the teleological framing—whereby a species’ milk is assumed to have been selected to produce a particular effect in its own offspring—that can accompany such comparisons. These signatures are best read as prioritized hypotheses about where species differences in milk regulatory potential, if real, would be concentrated.

### 3.4. The Dietary Bioavailability Question

The interpretation of every result above is conditional on the unresolved question of whether dietary milk miRNAs are absorbed and active in the consumer. Proponents have reported absorption of bovine-milk miRNAs and exosomes with measurable downstream effects, and cellular uptake of milk exosomes by multiple human cell types [14,15,16,17], while skeptics have found limited evidence for biologically meaningful uptake in adults and have raised concerns about quantification and endogenous contributions [21,22,23]; balanced reviews regard the question as open [24,25]. Our study does not adjudicate this debate. Rather, it assumes uptake as a modeling premise in order to map, conditionally, which host pathways would be most engaged and how this would differ by milk species. The exceptional stability conferred by vesicular encapsulation [28] makes the scenario most relevant to raw or minimally processed milk, in which intact exosomes are more likely to be preserved; processing such as pasteurization and homogenization may alter EV integrity and cargo and would be an important variable in any exposure-relevant study. We therefore present the targetome explicitly as a hypothesis-generating prioritization, contingent on bioavailability that future work must establish.

This sensitivity to handling has both a practical and a conceptual consequence. Practically, the direct miRNA-mediated effects modeled here would be preserved only when milk reaches the consumer with its extracellular vesicles intact—favoring raw or gently processed milk maintained under cold-chain conditions—whereas pasteurization, homogenization, prolonged storage, and repeated freeze–thaw degrade EV-associated miRNA, with reported losses of a large fraction of individual miRNAs after standard dairy processing [18,28,46]; where the cargo is substantially degraded, any direct regulatory effect would be attenuated accordingly. Conceptually, however, the value of a species-resolved miRNA map is not exhausted by direct targeting. We hypothesize, speculatively, that the miRNA spectrum—its overall shape and the relative dominance of particular families—may co-vary with other quantitative compositional features of a given milk, such as its protein, lipid, oligosaccharide, and metabolite profiles, because these all arise from the same species-specific mammary physiology. On this view, the species-specific miRNA fingerprint could serve as a compact molecular proxy for broader between-species compositional differences, retaining comparative and analytical utility even under conditions in which the miRNAs themselves are no longer bioactive. This proposal is offered strictly as a hypothesis for future testing.

### 3.5. Relationship to Prior Work

Our abundance spectra are consistent with prior single-species and comparative milk miRNome studies in human [5,32,47,48,49], ruminant [29,33,34,50,51], and other mammalian milks [52], including the consistent prominence of miR-148a and let-7 [53]. The added value of the present work is integrative and comparative: rather than describing one milk’s miRNAs, we project four milks’ abundance-weighted cargo onto a shared human-target space and formally separate conserved from divergent targeting. Earlier in silico efforts examined a single donor species or individual candidate miRNAs, typically using sequence-complementarity prediction alone and weighting each miRNA equally—for example, screening the bovine miRNome against human mRNAs [54] or tracing single digestion-resistant miRNAs to human pathways [52]. Our design differs on three axes that constitute its principal methodological contribution: it spans the four most consumption-relevant milks simultaneously; it weights predicted targeting by exosomal miRNA abundance, so that the most abundant cargo contributes proportionately; and it combines predicted (mirDIP) with experimentally supported (TarBase v9) interactions, mitigating the noise inherent to any single prediction tool [55]. This enables the formal separation of a conserved pan-milk core from species-restricted signatures and the confidence–exclusivity analysis, neither of which is available from single-species catalogues. Prior functional studies of milk EVs have emphasized immunomodulatory and anti-inflammatory activities [3,29,56], which is consistent with the immune-related targets that recur in our donkey-specific and core categories. To our knowledge, the systematic, category-resolved cross-species targetome reported here, together with the protocadherin-cluster and RNAi-machinery observations, has not been described previously.

### 3.6. Limitations

This study has substantial limitations that constrain interpretation, and we state them plainly. First and most importantly, the analysis is computational: targeting scores are predictions integrating database evidence, not measurements of repression in any tissue, and no experimental validation is presented or implied. Second, the weighting scheme is a deliberate simplification that captures the presence and relative abundance of targeting miRNAs rather than absolute molecular stoichiometry, miRNA–target affinity, target-site number, or competition among transcripts; it should be read as a relative prioritization, not an absolute effect size. Third, abundances are relative and presence-based; we did not model absolute copy number, dose, or the threshold abundance required for biological effects. Fourth, the sample sizes are modest and unevenly distributed (notably goat, *n* = 4), and milk miRNA content is known to vary with diet, breed, lactation stage, sample handling, and library preparation—sources of variation we could not fully control by aggregating public data, although using independent cohorts per species mitigates single-study artifacts.

We also note that the source datasets sampled different lactation stages and, in some cases, colostrum rather than mature milk. Nonetheless, the rank and relative distribution of the dominant conserved miRNAs were broadly concordant across datasets, suggesting that the species-level miRNA spectrum is relatively robust to milk state, and our comparison in any case concerns differences between species rather than within-species variation in milk stage. Fifth, quantifying all reads against human references restricts the analysis to conserved miRNAs with human orthologs and necessarily omits lineage-specific miRNAs; this is appropriate for modeling effects on the human transcriptome but underestimates total species divergence. Sixth, the fold-change threshold of 1.5 and the noise filter are user-defined choices that influence category boundaries; we therefore verified that the category architecture is stable across a range of thresholds and that the species-specific structure exceeds a permutation null (Section 2.10, Figure 5), and we note that the genome-referenced enrichment of the conserved core is partly subject to the known target-set bias of over-representation testing [42]. Seventh, the near-absence of goat-exclusive genes is expected by construction rather than being evidence about biology: because the cow and goat miRNomes are closely related, most goat targeting is shared with cow and is absorbed into the Cow_Goat_Specific category, and a strict per-species gap rarely isolates goat from cow; the modest goat sample size reinforces but does not principally cause this, so goat-exclusive signals should not be over-interpreted. Eighth, experimental-support counts (TarBase) are biased toward well-studied genes, which can inflate the apparent prominence of intensively researched targets such as *AGO2*. Finally, we modeled the canonical repressive mode of miRNA action and interpreted high targeting scores as predicted down-regulation of the target gene; we did not model the rare instances of miRNA-mediated activation, and we deliberately did not infer the direction of net pathway output, which depends on whether the targeted nodes are positive or negative regulators. We also did not model 3′-UTR polymorphisms, isoform or strand selection, or recipient-cell context, all of which would modulate any real regulatory outcome.

The number of samples was unequal across species (human 8, cow 13, goat 4, donkey 4). We mitigated this by comparing abundance-weighted per-species spectra rather than per-sample values and by testing the category structure against a permutation null, and samples nonetheless separated cleanly by species (Section 2.11); still, the smaller goat and donkey cohorts yield less stable spectra, and their species-specific sets should be regarded as more provisional.

The datasets also differ in milk state (mature milk, colostrum, and milk fractions), isolation method, and platform (Supplementary Material S10). Although the species signal dominated this technical variation in our clustering analysis (Section 2.11), we cannot exclude residual batch effects on individual low-abundance miRNAs, and we therefore restrict interpretation to the dominant, conserved spectrum rather than to individual rare species.

Finally, and most fundamentally, the milk samples compared here were not obtained under a common, controlled sampling design: they were derived from independent studies with differing and largely undocumented animal breeds, individual genetics, lactation management, and diets. The cross-species differences we report may therefore be partly confounded by these uncontrolled breeding, individual and dietary influences rather than reflecting species biology alone. For this reason, the present work should be regarded as a preliminary, hypothesis-generating analysis of publicly available data, and the species-specific patterns it proposes will require confirmation in controlled, standardized experiments in which breed, diet, lactation stage, and sample handling are matched across species.

### 3.7. Future Directions

The principal utility of this work is as a prioritized resource. The conserved core identifies candidate pan-milk targets and the self-referential RNAi-machinery hypothesis for mechanistic testing; the species-specific signatures identify where milk-type differences, if biologically real, would be concentrated, including the donkey immunoregulatory module and the bovine-excluded protocadherin cluster. We encourage the use of these data (Supplementary Materials S1–S11) as a foundation for targeted experiments: quantification of absolute miRNA copy numbers in defined EV preparations; controlled in vitro and organoid uptake and reporter assays; animal feeding studies using raw versus processed milk; and integration with matched proteomic and methylomic readouts to test the predicted epigenetic axis. Such studies would convert the predictions catalogued here into testable, and potentially actionable, biology.

Turning these predictions into evidence will require a defined experimental program, which the present resource is designed to prioritize. A tractable sequence would be the following: (i) quantitative confirmation (RT-qPCR or droplet-digital PCR) of the dominant conserved miRNAs—miR-148a-3p and the let-7 family—in independently collected, EV-purified milk of each species, to establish that the abundance ranking underlying our scores is reproducible; (ii) uptake assays in physiologically relevant recipient cells (for example intestinal epithelial lines such as Caco-2 or HT-29 and primary macrophages) using fluorescently labelled species-specific milk EVs, to test whether the cargo reaches the cytoplasm at functionally relevant doses [57]; (iii) direct target-repression assays for the highest-priority core interactions identified here—3′-UTR luciferase reporters for *DNMT1* (miR-148a) and for the convergence hub *ZEB2* (miR-200 family), combined with miRNA mimic/inhibitor transfection and measurement of the endogenous target; and (iv) functional read-outs in recipient cells and, ultimately, dose-controlled in vivo feeding studies, reading out the pathway-level consequences predicted here (for example DNA-methylation changes downstream of *DNMT1* repression, or epithelial–mesenchymal markers downstream of *ZEB2*). Only such experiments can establish whether the predicted targetome corresponds to real regulation; our contribution is to make that program specific and prioritized rather than open-ended.

Beyond milk, the abundance-weighted, integrated-evidence, cross-source targetome framework introduced here is not specific to any one source and could be applied to other dietary or therapeutic sources of extracellular-vesicle miRNA—other animal milks, and potentially plant-derived exosome-like nanoparticles that have attracted interest as oral delivery vehicles. An important methodological caveat is that plant miRNAs cannot be quantified against a human reference: they lack human orthologs, and carry a 3′-terminal 2′-O-methylation and other structural distinctions, and their cross-kingdom uptake and activity in mammals remain debated [13]. Extending the approach to such sources would therefore require sequence-based cross-kingdom target prediction against the human transcriptome rather than reference mapping, together with the same caution about bioavailability emphasized throughout this work.

## 4. Materials and Methods

### 4.1. Data Acquisition

Publicly available small-RNA sequencing datasets of milk and milk-derived cells/EVs were obtained for four species. Human samples (*n* = 8) comprised breast-milk exosome libraries (SRR346516–SRR346519; BioProject PRJNA147351) and milk miRNA-seq libraries (SRR2976280, SRR2976297, SRR2976303, SRR2976307; PRJNA305166), corresponding to published human milk miRNA studies [5,32,58]. Cow samples (*n* = 13) comprised milk-EV libraries from PRJNA771627 (SRR16352778/779/782) and ten additional bovine colostrum libraries from PRJNA1165141 [59]. Goat samples (*n* = 4) comprised SRR16352790–792 (PRJNA771627) and SRR29674790 (PRJNA1130716). Donkey samples (*n* = 4) comprised SRR16352793–795 (PRJNA771627) and SRR29674788 (PRJNA1130716). The ruminant and donkey EV datasets correspond to published comparative milk-EV studies [29,33,34]. Using independent cohorts per species was intended to reduce single-study technical bias; only datasets with abundance spectra consistent with well-prepared milk small-RNA libraries (e.g., expected prominence of miR-148a and let-7 family members) were retained, since post-sequencing processing quality varies substantially across public datasets. Datasets were further required to deposit raw small-RNA sequencing reads (SRA/GEO) rather than only processed results, so that every sample could be reprocessed uniformly through the same pipeline; we judged this cross-study consistency more important than maximizing the number of included studies. The provenance and available characteristics of every dataset—species, milk type or fraction, lactation stage or parity, breed, health status, EV/RNA isolation, and sequencing platform—are summarized in Supplementary Material S10, where an attribute was not stated in the primary study it is marked “not reported”.

### 4.2. microRNA Quantification and Reference Mapping

Reads were processed and quantified using an established miRNA identification and quantification approach based on the miRDeep2 framework [35] (version 2.0.1.3), with mature miRNA annotations from the miRBase database [36] (release 22.1; University of Manchester, Manchester, UK). All samples, regardless of species, were quantified against human (hsa) miRBase references. This design choice was intentional: because the objective is to model the potential effect of milk miRNAs on the human transcriptome, mapping to human references focuses the analysis on conserved miRNAs that possess human orthologs and can be assigned human target genes, at the explicit cost of excluding species-specific miRNAs lacking a human counterpart (Section 3.6). This approach is biologically reasonable because the most abundant milk miRNAs belong to deeply conserved seed families whose mature sequences are essentially identical across mammals, so the dominant cargo—for example miR-148a-3p and the let-7 family—maps to human references with high fidelity [7,9]. We also deliberately restricted quantification to annotated miRBase miRNAs and did not attempt de novo discovery of novel or unannotated species, because such candidates lack human orthologs and predicted or validated human targets (and so cannot be assigned target genes through mirDIP or TarBase), are typically of low abundance and therefore contribute little to an abundance-weighted analysis, and are often dataset-specific and less reproducible. For each sample, mature-miRNA counts were normalized to relative abundance (percentage of total mapped miRNA reads).

Across the 29 libraries, the proportion of cleaned reads aligning to the human miRBase reference ranged from 0.85% to 76.6%. Alignment was highest for the PRJNA771627 cow, goat and donkey libraries (59.5–76.6%), intermediate for the human libraries (median ≈ 54%, range 30.8–68.4%) and for the PRJNA1165141 cow libraries (33.0–65.9%), and very low for the two PRJNA1130716 cross-species libraries (6.68% for donkey SRR29674788 and 0.85% for goat SRR29674790). These last two libraries yielded few human-mappable reads and therefore contributed little to the targetome; their poor alignment is expected, because quantification against human references by design retains only conserved miRNAs with human orthologs, and it is consistent with the goat under-representation noted in the results (Section 2.4 and Section 3.6). To confirm that these two low-yield libraries do not drive our findings, we recomputed the per-species abundance spectra after excluding them; the dominant spectra were essentially unchanged (Spearman ρ = 0.99 for both goat and donkey), and miR-148a-3p remained the most abundant miRNA in every species. Full per-sample sequencing depths and library-preparation details are reported in the original data publications [5,29,32,33,34]; the alignment rates reported here were obtained in the present reanalysis.

### 4.3. Abundance Spectra and Pooling

Per-sample relative abundances were summarized to per-species spectra (Supplementary Material S1) and retained at the per-sample level (Supplementary Material S2). A pooled cross-milk ranking was computed as the average relative abundance of each miRNA across milks (Supplementary Material S3), from which cumulative-abundance statistics and the shared-miRNA count were derived. Descriptive summaries (top-N cumulative fractions; number of miRNAs shared by all four species) were computed directly from these tables.

### 4.4. Target Prediction and Experimental-Support Integration

Predicted human targets of each detected miRNA were retrieved from the microRNA Data Integration Portal (mirDIP; version 5.2; University of Toronto, Toronto, ON, Canada), which aggregates multiple target-prediction resources into an integrated confidence score and ranking [37,38]; only interactions in the high and very-high mirDIP confidence classes were retained for scoring. Experimentally supported miRNA–gene interactions, together with the number of supporting experiments, were retrieved from TarBase v9 (DIANA-Tools, University of Thessaly, Volos, Greece) [39]. Although mirDIP already incorporates experimental evidence in its integrative ranking, the number of mirDIP predictions and the number of TarBase experiments were retained as separate, explicit columns to support transparent interpretation and to allow readers to distinguish predicted from experimentally documented interactions.

### 4.5. Weighted Targeting Score

For each gene g and species s, the weighted targeting score was computed as an abundance-weighted, confidence-scaled sum over all miRNAs that target g and are present in species s (Equation (1)):

*score*_*g*,*s*_ = Σ_*m*_
*a*_*m*,*s*_ (*r*_*m*,*g*_ + *b*_*m*,*g*_) (1)

where the sum runs over every miRNA m detected in species s that is predicted to target g; *a*(*m*,*s*) is the mean relative exosomal abundance of m in species s (its percentage of mapped miRNA reads); *r*(*m*,*g*) is the mirDIP integrated confidence of the m–g interaction; and *b*(*m*,*g*) is a fixed validation bonus equal to 0.5 when the same pair is also documented experimentally in TarBase v9, and 0 otherwise. Only miRNAs detected in a given milk contribute to that milk’s score, so a gene can receive different scores across species purely through differences in miRNA abundance, even when the underlying predicted interactions are shared. Because the leading term is relative abundance, the score reflects primarily the presence and relative abundance of targeting miRNAs rather than absolute molecular stoichiometry, target-site number, or miRNA–target affinity, and should be read as a relative prioritization of targeting pressure rather than a quantitative prediction of repression magnitude; in this dataset, the resulting values span approximately 0–100. The result is a four-value vector (human, cow, goat, donkey) per gene; the complete matrix for all targeted genes is provided in Supplementary Material S6 (a summary sheet with all four per-species scores, support counts, and an average targeting score, plus per-species sheets ranked by score) and the categorized retained subset in Supplementary Material S4. Weighting predicted targeting by relative exosomal abundance reflects a simple biological rationale: a more abundant miRNA delivers more copies per unit of ingested EV cargo and therefore has greater potential to occupy target sites, whereas a rare miRNA—however high-confidence its predicted pairing—contributes little to the aggregate regulatory pressure on a gene. This abundance dependence is consistent with experimental evidence that dietary milk-EV miRNAs require a minimum delivered dose to measurably affect recipient gene expression [57].

### 4.6. Gene Filtering and Categorization

To remove weakly or non-specifically targeted genes, a noise filter was applied: a gene was discarded if its summed cross-species score was below 10 or if its maximum single-species score fell below T, defined as the 60th percentile of all non-zero targeting scores. This removed 6039 genes and retained 4577 (the complete pre-filter matrix of all 10,616 targeted genes is provided in Supplementary Material S6, and the retained, categorized set in Supplementary Material S4). Each retained gene was then categorized from its four per-species scores: the scores were ranked in descending order and scanned for the first adjacent pair whose ratio was at least 1.5 (the fold-change threshold), which marks the boundary separating a dominant group (all species above the gap) from the remainder. The dominant group defined the category name (e.g., Human_Specific, Cow_Goat_Specific). When no such gap existed, the gene was labeled Core_Pan_Milk if its minimum score was at or above T (uniformly high targeting) and Variable_Gradient otherwise (a smooth gradient without discrete boundaries). For reporting in Table 2 and Table 3, a per-gene FC value was additionally computed as the mean score of the dominant group divided by the highest-scoring non-dominant species (Equation (2)):

*FC_g_* = (mean*_s_*_∈*D*_ *score_g_*_,*s*_)/(max*_s_*_∉*D*_ *score_g_*_,*s*_) (2)

where D is the dominant group of species for gene g. This categorization was designed for enrichment analysis and for detecting pronounced cross-species deviations; assignment to a category does not imply that a gene is targeted exclusively by that milk, only that it is most strongly influenced by the defining species. For each category, the fifty top-ranked genes were compiled for focused analysis (Supplementary Material S5).

### 4.7. Functional Enrichment

Gene Ontology [60,61] biological-process and KEGG [62,63] pathway over-representation analyses were performed on each gene set with Enrichr [64,65] (Ma’ayan Laboratory, Icahn School of Medicine at Mount Sinai, New York, NY, USA) through its Python interface in the GSEApy package (version 1.1.3) [66], querying the GO_Biological_Process_2023 and KEGG_2021_Human libraries against the human gene universe; sets with fewer than ten genes were not tested. Two complementary analyses were run: a per-category analysis on each categorized set (Supplementary Material S7) and a per-milk “global” analysis on all genes with a targeting score of at least 5 for that milk (Supplementary Material S8). Terms with a nominal *p* of <0.05 were retained (the top fifteen per category and top twenty per milk). To incorporate abundance weighting, each enriched term was additionally assigned a cumulative regulatory impact (CRI) equal to the sum of the targeting weights of its member genes (Equation (3)):

CRI*_t_* = Σ*_g_*_∈*G*_ *w_g_* (3)

where the sum runs over the genes g annotated to term t (the analyzed gene set G), and the weight w of each gene is its mean score across the dominant species for the per-category sets, or that milk’s own score for the per-milk global sets; terms were ranked by this weighted impact. The representative summary in Figure 4 was redrawn for clarity from these results, retaining the Enrichr significance values. All enrichment-derived functional themes are reported as computational predictions.

### 4.8. Visualization and Statistics

Figures were generated in Python (version 3.11.7; Python Software Foundation, Wilmington, DE, USA) using the matplotlib (version 3.8.2), seaborn (version 0.13.0), NumPy (version 1.26.2), pandas (version 2.1.4), and SciPy (version 1.11.4) libraries with a colorblind-accessible palette. The analysis is primarily descriptive—relative abundances, weighted scores, fold changes, and enrichment categories are reported without modeling effect sizes—with two robustness analyses. First, the categorization was repeated across fold-change thresholds of 1.3–3.0 and across noise-filter settings (50th–70th score percentile; minimum summed score 5–20) to confirm that the category architecture is not an artifact of specific parameter choices (Figure 5A; Supplementary Material S9). Second, to test whether the species attribution of targeting exceeds chance, the four per-species scores were permuted within each gene (1000 permutations) and the categories re-derived, giving an empirical null distribution for each category size against which the observed size was compared (one-sided empirical *p*; Figure 5B). Cross-species similarity of the weighted scores was summarized by Spearman correlation over the retained genes (Figure 5C). We note that standard over-representation testing is known to be biased for miRNA target sets, which are enriched for genes with many target sites independently of biological condition [42]; the enrichment results are interpreted with this caveat, and we place greater weight on between-category (species-versus-species) contrasts than on the genome-referenced significance of the conserved core.

## 5. Conclusions

This comparative in silico analysis indicates that the milk exosomal miRNA targetome of human, cow, goat, and donkey milk is organized around a large, conserved pan-milk core—dominated by miR-148a and let-7 and enriched for transcriptional regulation, growth signaling and, notably, the RNA-interference machinery itself—upon which species-specific signatures are superimposed, including a human neuronal/chromatin bias, a donkey transcriptional/immune bias, and a ruminant lipid/insulin–mTOR module. Across categories, a reproducible confidence–exclusivity trade-off links a signature’s specificity to the abundance of the miRNAs that define it. Because miRNAs repress their targets, these maps are best read as predictions of which recipient genes would be down-regulated—clearly at the gene level (for example, miR-148a-predicted repression of *DNMT1*), while net pathway direction remains gene-sign-dependent and is left open. These results are computational predictions that assume dietary miRNA uptake and do not constitute experimental validation; their value lies in providing a structured, prioritized and fully documented resource (Supplementary Materials S1–S11) to guide future functional, nutritional, and epigenetic studies of milk miRNA bioactivity and of how the choice of milk species might shape that bioactivity.

## Figures and Tables

**Table 1:** Overview of the sixteen targetome categories. Gene counts and percentages are computed over the 4577 retained genes (Supplementary Material S4). Mean dominant score and mean fold change (FC) are computed over the top-50 representative genes per category (Supplementary Material S5); the dominant score for the conserved core reflects balanced cross-species targeting. Predominant predicted functional themes summarize the enrichment results (Supplementary Materials S7 and S8) and are computational predictions, not experimentally confirmed functions. Abbreviations: PI3K, phosphoinositide 3-kinase; MAPK, mitogen-activated protein kinase; TGF-β, transforming growth factor beta; EMT, epithelial–mesenchymal transition.

Category	*n*	% of 4577	Mean Dom./Mean FC	Predominant Predicted Functional Themes
Core_Pan_Milk	1809	39.5	74.8/—	Transcriptional regulation; PI3K–Akt, MAPK, TGF-β/SMAD signaling; autophagy; RNAi machinery
Donkey_Specific	770	16.8	44.2/1.74	Transcriptional/EMT control; retinol and xenobiotic metabolism; immune regulation (CD47)
Human_Specific	430	9.4	33.0/1.78	Axonogenesis and axon guidance; glutamatergic synapse; chromatin remodeling
Cow_Donkey_Goat_Specific	357	7.8	45.5/1.79	Shared non-human (ruminant + donkey) metabolic and signaling targets
Cow_Goat_Specific	296	6.5	45.0/1.65	Lipid/cholesterol metabolism; insulin–mTOR signaling; cell cycle
Variable_Gradient	187	4.1	10.6/—	Graded cross-species targeting; mixed regulatory functions
Cow_Goat_Human_Specific	156	3.4	40.1/1.79	Shared metabolic/transcriptional targets excluding donkey
Donkey_Goat_Human_Specific	129	2.8	15.9/2.63	Clustered protocadherin-α; calcium/lipid; chromatin (PRC2)
Donkey_Goat_Specific	121	2.6	19.7/2.15	Epithelial barrier and cytoskeleton (CTNND1, MACF1, CDC42)
Cow_Specific	98	2.1	12.2/1.66	Transcriptional/chromatin regulation (NR4A2, EZH2, ATF1/2)
Cow_Human_Specific	68	1.5	12.6/1.87	Insulin/IGF metabolism (GRB10); taurine transport (SLC6A6)
Donkey_Human_Specific	64	1.4	18.7/1.85	m6A modification (WTAP); lipid/mTOR–FOXO longevity axis
Cow_Donkey_Specific	51	1.1	11.5/1.97	Shared ruminant–donkey regulatory targets
Cow_Donkey_Human_Specific	38	0.8	11.0/2.18	RISC core (AGO2); mRNA destabilization; chromatin readers
Goat_Human_Specific	2	<0.1	8.1/1.96	Sparse; not interpreted (data-limited)
Goat_Specific	1	<0.1	16.0/1.51	Single gene (RELN); not interpreted (data-limited)

**Table 2:** Representative genes of the conserved pan-milk core. Weighted targeting scores (approx. 0–100) are shown for human (Human Score, H), cow (Cow Score, C), goat (Goat Score, G), and donkey (Donkey Score, D); “TarBase N.” is the number of supporting experiments recorded in TarBase v9 [39]. Annotated functions are from standard gene annotation and are provided for orientation only. Full values for the top fifty core genes are in Supplementary Material S5.

Gene	Human Score	Cow Score	Goat Score	Donkey Score	TarBase N.	Annotated Function (Orientation)
LCOR	77.7	98.3	90.7	74.2	1181	Transcriptional corepressor
ATP2A2	82.2	85.4	85.7	74.8	1265	ER calcium ATPase (SERCA2)
RNF38	76.0	87.9	93.3	91.5	220	RING-type E3 ubiquitin ligase
CHD9	78.0	90.7	90.5	88.4	818	Chromatin remodeling ATPase
CCNT2	63.7	82.7	92.7	92.4	851	Cyclin T2; transcriptional elongation
QKI	74.1	79.2	89.0	83.6	857	RNA-binding regulator
TNRC6B	62.9	76.5	74.6	81.5	1194	GW182 paralog; RISC effector
NUFIP2	59.4	69.1	73.0	75.2	1558	RNA-binding protein
CPEB4	72.3	89.4	80.2	71.4	681	Cytoplasmic polyadenylation control
FRS2	82.9	80.2	86.7	88.6	693	FGF receptor signaling adaptor
PTEN	52.6	72.5	77.8	79.8	637	PI3K–Akt pathway tumor suppressor
TGFBR1	71.9	70.5	69.8	71.2	608	TGF-β type I receptor
SMAD2	71.2	81.0	77.2	52.9	364	TGF-β/SMAD transcription factor
USP47	76.4	86.6	84.2	79.5	476	Deubiquitinating enzyme
CPEB3	67.7	86.6	87.5	87.4	458	Translational repressor

**Table 3:** Representative signature genes by category. “Dom. Score” is the dominant-species weighted targeting score; “FC” is the dominant-versus-rest fold change; “TarBase N.” is the number of supporting experiments in TarBase v9. Predicted functions are provided for orientation and reflect in silico inference, not experimental validation. Complete per-category lists are in Supplementary Material S5. Abbreviations: Dom. Score, dominant-species score; FC, fold change; LDL, low-density lipoprotein.

Category	Gene	Dom. Score	FC	TarBase N.	Predicted Function/Interpretation (In Silico)
Human_Specific	NR4A3	23.3	5.44	69	Immediate-early nuclear receptor; sharpest human-exclusive signal
Human_Specific	POU2F1	44.3	1.77	942	Transcription factor; neuronal/immune programs
Human_Specific	GRM5	30.2	2.42	142	Metabotropic glutamate receptor; glutamatergic synapse
Donkey_Specific	CD47	46.9	2.34	221	“Don’t-eat-me” immune signal; phagocytosis control
Donkey_Specific	PLAG1	59.9	1.75	419	Growth-associated transcription factor
Donkey_Specific	TET1	52.8	1.73	232	DNA demethylase; epigenetic regulation
Cow_Goat_Specific	LDLR	39.7	1.76	654	LDL receptor; cholesterol uptake
Cow_Goat_Specific	RPS6KB1	50.5	1.61	526	mTOR effector kinase; growth signaling
Cow_Goat_Specific	IRS1	43.5	1.58	519	Insulin receptor substrate
Donkey_Goat_Specific	CTNND1	—	4.01	441	Adherens-junction stability; epithelial barrier
Donkey_Goat_Specific	MACF1	36.0	2.19	1432	Cytoskeletal cross-linker
Donkey_Goat_Human	PCDHA cluster	—	≈3.2	—	14 clustered α-protocadherins; bovine-excluded (novel)
Donkey_Goat_Human	ZBTB43	—	5.79	—	Zinc-finger factor; highest FC in dataset
Cow_Donkey_Human	AGO2	13.8	1.96	1940	RISC catalytic core; highest experimental support
Cow_Human_Specific	GRB10	20.2	1.92	—	Imprinted IGF/insulin-signaling adaptor
Donkey_Human_Specific	WTAP	26.6	1.91	—	m6A methyltransferase component

## Data Availability

All primary data analyzed in this study are publicly available from the NCBI Sequence Read Archive and Gene Expression Omnibus under accessions PRJNA147351, PRJNA305166, PRJNA771627, PRJNA1130716, and PRJNA1165141, and GEO series GSE32253, GSE75726, and GSE227559. Target predictions were obtained from mirDIP (https://ophid.utoronto.ca/mirDIP/, accessed on 10 June 2026) and experimentally supported interactions from TarBase v9 (https://dianalab.e-ce.uth.gr/tarbasev9, accessed on 10 June 2026). The derived targetome and all summary tables are provided in Supplementary Materials S1–S11. All analysis code and the intermediate files needed to reproduce the figures are openly available on GitHub (maksimkamekspeks/milk-mirna-targetome).

## Supplementary Materials

The following supporting information can be downloaded at: https://www.mdpi.com/article/10.3390/ijms27156952/s1.
